# Studying the trend of the novel coronavirus series in Mauritius and its implications

**DOI:** 10.1371/journal.pone.0235730

**Published:** 2020-07-10

**Authors:** Naushad Mamode Khan, Ashwinee Devi Soobhug, Maleika Heenaye-Mamode Khan

**Affiliations:** 1 Department of Economics and Statistics/Faculty of Social Sciences and Humanities, University of Mauritius, Réduit, Mauritius; 2 Department of External Trade/Statistics Mauritius, Ministry of Finance, Economic Planning and Development, Port-Louis, Mauritius; 3 Department of Software and Information Systems/Faculty of Information and Communication Technologies, University of Mauritius, Réduit, Mauritius; University of Lincoln, UNITED KINGDOM

## Abstract

Mauritius stands as one of the few countries in the world to have controlled the current pandemic, the novel coronavirus 2019 (COVID-19) to a significant extent in a relatively short lapse of time. Owing to uncertainties and crisis amid the pandemic, as an emergency announcement, the World Health Organization (WHO) solicits the help of health authorities, especially, researchers to conduct in-depth research on the evolution and treatment of COVID-19. This paper proposes an integer-valued time series model to analyze the series of COVID-19 cases in Mauritius wherein the corresponding innovation term accommodates for covariate specification. In this set-up, sanitary curfew followed by sanitization and sensitization campaigns, time factor and safe shopping guidelines have been tested as the most significant variables, unlike climatic conditions. The over-dispersion estimates and the serial auto-correlation parameter are also statistically significant. This study also confirms the presence of some unobservable effects like the pathological genesis of the novel coronavirus and environmental factors which contribute to rapid propagation of the zoonotic virus in the community. Based on the proposed COM-Poisson mixture models, we could predict the number of COVID-19 cases in Mauritius. The forecasting results provide satisfactory mean squared errors. Such findings will subsequently encourage the policymakers to implement strict precautionary measures in terms of constant upgrading of the current health care and wellness system and re-enforcement of sanitary obligations.

## Introduction

Four months since the start of the COVID-19 outbreak, most affected states have imposed strict confinement measures, largely disrupting the daily sequence of works in various spheres. Most people are working from home with limited resources, online teaching instead of traditional classroom teaching are being conducted and strict sanitization measures in public places, especially in super, and hypermarkets have been imposed. Overall, the world is in an unexpected turmoil. However, there is still a ray of hope, especially, when developing sub Saharan African countries with limited economic resources, like Mauritius and Seychelles, are combating the pandemic with full vigor.

Setting the focus on Mauritius, this is one of the countries whose economy has made great strides and as of the end of April 2020, the health authorities officially reported that more than 90 percent of COVID-19 patients have recovered (312 recovered cases out of 332 confirmed cases, with 7 active cases). Indeed the situation was alarming when the first cases of COVID-19 was reported on the 18th March 2020 and within four weeks, the total number of confirmed cases climbed to 325, an exponential increase, and the total number of deaths was hovering at 9. Considering the severity of the situation, policy makers announced a six-week national complete lockdown, effective from the 20th March 2020, high-tech medical diagnostic apparatus, key protective hygiene and sanitation equipment and life saving medical drugs like hydroxychloroquine tablets, worth a colossal amount, were purchased and even obtained free of charge from sister countries like India, to upgrade the quality of health services and fight the pandemic. Thorough COVID-19 screening is being conducted on a daily basis. Within one month, by the end of April 2020, around 17,196 polymerase chain reaction (PCR) tests in addition to 19,393 rapid swab tests have been conducted among “frontliners” who are hospital health care workers and essential service workers, representing around 3 per cent of the whole population. This measure is indeed applaudable as by early detection of the virus, some patients can be cured quickly. As pro-active measures, Mauritius is better equipped with an average of 3.4 hospital beds per 1000 population, as compared to developed countries like the United Kingdom (UK) and other Sub Saharan countries [1] and with the collaboration with private stakeholders, an adequate number of quarantine centers have been instituted. All these measures, along with the positive changes in the population behaviour in regard to confinement, have altogether helped to contain the pandemic in Mauritius.

The measures adopted by Mauritius have highly been praised by international organizations such as World Health Organization (WHO) and United Nations (UN). As a retrospective, it is important to retrieve, via inferential statistical methods, the factors that have contributed significantly in controlling the propagation of the novel coronavirus in Mauritius. Besides, since the COVID-19 data is in the form of an integer-valued time series [2] layout, it is of research importance to develop an integer-valued regression time series process that can provide insights on the significance of explanatory variables as well set a platform to forecast the possible number of COVID-19 infected cases which is quite tedious as illustrated in [3, 4, 5]. Admittedly, research findings in [6] indicate that the COVID-19 data exhibits lots of dynamic and latent features and hence, to cater for such volatilities, we introduce random effects in our model specification. In this context, we propose Gamma [7] and Normal [8] random effects that, in addition to the covariate specifications, would render the model estimators less biased and improve the forecasts. In the same vein, such forecasts will aid policymakers in articulating post pandemic measures, since Mauritius, which is highly dependent on the hospitality sector, is expected to face a drastic contraction in its economic growth. The research findings in this paper will evidently guide policy makers and add value to the limited research undertaken so far in the case of COVID-19 in Mauritius. It is important to understand the evolution of COVID-19 in Mauritius otherwise as an import-dependent country, Mauritius may find itself engulfed in the darkness of COVID-19 in terms of loss in economic wealth and deterioration in population health state.

The paper therefore proposes an integer-valued auto-regressive model (INAR(1)) with Conway-Maxwell Poisson (COM-Poisson) mixed innovation terms that can accommodate for covariate, random effect and serial auto-correlation specifications. The organization of the paper is as follows: In Section 2, material in terms of time series of COVID-19 in Mauritius and proposed methods principally the INAR(1) Process and the COM-Poisson mixture innovations have been provided. Estimation of parameters are in Section 2 as well. In Section 3, the results have been interpreted followed by some discussions in Section 4. Finally, Section 5 concludes the study.

## Methods

The local data on COVID-19 is publicly available in European Union Open Data Portal at S1. As aforementioned, Mauritius registered its first three cases on the 18th March 2020 and as at 11th April 2020 or after 23 days, the health authorities of Mauritius officially reported 318 active cases of COVID-19 and 7 deaths. This signifies an overall average of 13.8 COVID-19 cases per day with a variance of 102.2. Detailed statistics for the 23 days are given at S2. Currently, three important peaks have been noted on Day 8 (25th March 2020), Day 18 (5th April 2020) and Day 22 (10th April 2020) where 33, 31 and 41 patients were tested positive for COVID-19 respectively. Based on these statistics, it is obvious that the data are exhibiting severe over-dispersion. This is confirmed by the *qcc.overdispersion.test* in R statistical software via the *qcc* package that yields a chi-squared p-value of 8.4477*e* − 13, showing a high level of significance. Moreover, the *rcorr* and the *adftest* illustrate that the data exhibit some serial correlation and are non-stationary. This indicates that the number of infected cases in Mauritius shows some fluctuations as well. The *VarSelect* routine in the library *Vars* showed that the Akaike Information Criterion (AIC) yielded a lag order of 1, and hence we can conclude that the model proposed in section 3 is suitable to model the above series.

Let *y*_*t*_ indicates the number of infected or COVID-19 positive case at the *t*^*th*^ time point, *t* = 1, 2, 3, …, *T* and $\tilde{y_{t}}=y_{t}|\gamma$, where *γ* describes the aforementioned latent effect. In the subsequent parts of this section, we show the different probability models for *γ*. Thus, the vector of time series conditional count observations is represented by $\tilde{y}={\left[\tilde{y_{1}},\tilde{y_{2}},\ldots ,\tilde{y_{t}},\ldots ,\tilde{y_{T}}\right]}^{{}^{\prime }}$. It is important to assume that the observations $\tilde{y_{t}}$ and $\tilde{y_{\left(t+h\right)}}$ are likely to be serially correlated, where the lag $h\in Z^{+},1\leq h\leq T-1$. Due to the latent effect *γ*, the covariance between the unconditional observation *y*_*t*_ and *y*_*t* + *h*_ is computed as:

$$\begin{array}{ccc} Cov(y_{t},y_{t+h}) & = & E\left[Cov\left(\tilde{y_{t}},\tilde{y_{t+h}}\right)\right]+Cov\left[E\left(\tilde{y_{t}}\right),E\left(\tilde{y_{t+h}}\right)\right] \end{array}$$

We further assume that the observation *y*_*t*_ is subject to a *p* × 1 vector of covariates, denoted by $x_{t}={\left[x_{t,1},x_{t,2},x_{t,3},\ldots ,x_{t,k},x_{t,(k+1)},\ldots ,x_{t,p}\right]}^{{}^{\prime }}$ that comprises of both time-variant and time-invariant exogeneous variates. The regression vector *β* is denoted by ***β*** = [*β*_1_, *β*_2_, …, *β*_*k*_, *β*_*k*+1_, …, *β*_*p*_]. As stated earlier, we assume that the conditional observation $\tilde{y_{t}}$ follows the INAR(1) process that satisfies the first-order auto-regressive equation [9]

$$\begin{array}{c} \tilde{y_{t}}=\rho *\tilde{y_{\left(t-1\right)}}+\tilde{r_{t}} \end{array}$$ (1)

where *r*_*t*_ is the random innovation component and *ρ* ∈ (0, 1) independent of the observation $\tilde{y_{t}}$, $Cov(\tilde{y_{t-k}},\tilde{r_{t}})=0$, for $k\in Z^{+}$ and * indicates the binomial thinning operator where $\rho *\tilde{y_{t-1}}|\tilde{y_{t-1}}∼Binomial\left(\tilde{y_{t-1}},\rho \right)$ [9]. Hence,

1. $E\left(\rho *\tilde{y_{t}}\right)=\rho E\left(\tilde{y_{t}}\right)$
2. $V\left(\rho *\tilde{y_{t}}\right)=\rho \left(1-\rho \right)E\left(\tilde{y_{t}}\right)+\rho^{2}V\left(\tilde{y_{t}}\right)$

The above auto-regressive equation can be re-written in terms of the error components *r*_*t*_ [10] as:

$$\begin{array}{ccc} \tilde{y_{t}} & = & \sum_{k=0}^{\infty }\rho^{k}*\tilde{r_{t-k}} \end{array}$$

and hence the moments of $\tilde{y_{t}}$ are derived from the distributional properties of $\tilde{r_{t}}$ and using the binomial thinning properties.

We now introduce the COM-Poisson mixture model, wherein the innovation term $\tilde{r_{t}}$ in Eqn(1) follows the COM-Poisson mixture as:

$$\begin{array}{c} f\left(\tilde{r_{t}}\right)=\frac{\tilde{\lambda_{t}}\tilde{r_{t}}}{{\left(\tilde{r_{t}}!\right)}^{\nu }}{\left[Z(\tilde{\lambda_{t}},\nu )\right]}^{-1} \end{array}$$ (2)

where $\tilde{r_{t}}=0,1,2,\ldots ,..$ and *ν* ≥ 0 s.t *ν*(<)>1 indicates (over)under-dispersion. The normalizing constant $Z\left(\tilde{\lambda_{t}},\nu \right)=\sum_{s=0}^{\infty }\frac{\tilde{\lambda_{t}^{s}}}{{\left(s!\right)}^{\nu }}$. Thus, it can be deduced that for *ν* = 1, $\tilde{r_{t}}$ follows the Poisson distribution with mean parameter $\tilde{\lambda_{t}}$, for *ν* = 0 and $\tilde{\lambda_{t}}<1$, $\tilde{\lambda_{t}}$ is Geometric with probability $1-\tilde{\lambda_{t}}$ and for *ν* → ∞, $\tilde{\lambda_{t}}$ is Bernoulli with probability $\frac{\tilde{\lambda_{t}}}{1+\tilde{\lambda_{t}}}$. Hence, the COM-Poisson generalizes some important discrete models and for some substitutions, the COM-Poisson acts a bridge between the Binomial, Geometric and Negative Binomial. However, in practice, the exact evaluation of $Z(\tilde{\lambda_{t}},\nu )$ is nearly impossible for a general *ν* > 0. The following approximation was suggested in [11]

$$\begin{array}{cc} Z(\tilde{\lambda_{t}},\nu ) & =\frac{\text{exp}(\nu {\tilde{\lambda_{t}}}^{\frac{1}{\nu }})}{{\tilde{\lambda_{t}}}^{(\nu -1)/2\nu }{\left(2\pi \right)}^{(\nu -1)/2}\sqrt{\nu }}\left(1+O\left({\tilde{\lambda_{t}}}^{-1/\nu }\right)\right) \end{array}$$

and this proves to yield satisfactory results especially for very large λ, as illustrated in [12, 13]. In fact, under this approximation, the moments of $\tilde{r_{t}}$ can be expressed suitably in closed forms as follows:

$$\begin{array}{cc} E\left(\tilde{r_{t}}\right)=\tilde{\lambda_{t}}\frac{d}{d\tilde{\lambda_{t}}}[Z(\tilde{\lambda_{t}},\nu )] & =\tilde{\mu_{t}}\approx {\tilde{\lambda_{t}}}^{1/\nu }-\frac{\nu -1}{2\nu },\\ V\left(\tilde{r_{t}}\right)=\tilde{\lambda_{t}}\frac{d}{d\tilde{\lambda_{t}}}E\left(\tilde{r_{t}}\right) & ={\tilde{\sigma^{2}}}_{t}\approx \frac{{\tilde{\lambda_{t}}}^{1/\nu }}{\nu }, \end{array}$$

whilst still, the above approximations satisfy the over-(under) dispersion assumptions for *ν* < (>)1. In fact, $\tilde{\lambda_{t}}=\text{exp}\left(x_{t}^{{}^{\prime }}\beta +\gamma \right)$. In the same vein, an alternative re-parameterization of the COM-Poisson was proposed in [14], by allowing $\tilde{\mu_{t}}=\text{exp}\left(x_{t}^{{}^{\prime }}\beta +\gamma \right)$. As for the model specification for *γ*, we let *W* = *W*(*κ*^2^) = exp(*γ*) follows the exponential of the respective probability function, assuming *κ*^2^ = *σ*^2^ + *ν*. The moments of the unconditional response, *y*_*t*_ are given as below and the detailed proof is in S1 Appendix:

1. $E\left(r_{t}\right)=\text{exp}\left({X_{t}}^{\prime }\beta \right)E\left(\text{exp}\left(\gamma \right)\right)$
2. $V\left(r_{t}\right)=\nu^{-1}[\text{exp}\left({X_{t}}^{\prime }\beta \right)E\left(\text{exp}\left(\gamma \right)\right)+\frac{\nu -1}{2\nu }]+\text{exp}\left(2{X_{t}}^{\prime }\beta \right)V\left(\text{exp}\left(\gamma \right)\right)$
3. $E\left(Y_{t}\right)=E\left(\text{exp}\left(\gamma \right)\right)\sum_{k=0}^{\infty }\rho^{k}\text{exp}\left({X_{\left(t-k\right)}}^{\prime }\beta \right)$
4. $\begin{array}{c} V\left(Y_{t}\right)=E\left(\text{exp}\left(\gamma_{i}\right)\right)\sum_{k=0}^{\infty }\rho^{k}\left(1-\rho^{k}\right)\text{exp}\left(X_{\left(t-k\right)}{}^{\prime }\beta \right)+\\ \sum_{k=0}^{\infty }\rho^{2k}\nu^{-1}\left[\text{exp}\left(X_{\left(t-k\right)}{}^{\prime }\beta \right)\times E\left(\text{exp}\left(\gamma \right)\right)+\frac{\nu -1}{2\nu }\right]+\sum_{k=0}^{\infty }\rho^{2k}\text{exp}\left(2X_{ij}{}^{\prime }\beta \right)\times V\left(\text{exp}\left(\gamma \right)\right) \end{array}$
5. $\begin{array}{l} Cov(y_{t},y_{\left(t+h\right)})=\rho^{h}\left(\sum_{k=0}^{\infty }\rho^{k}(1-\rho^{k})E(\text{exp}(\gamma ))\text{exp}(X_{\left(t+h-k\right)}{}^{\prime }\beta )\right)+\\ \rho^{h}\left(\sum_{k=0}^{\infty }\rho^{2k}\nu^{-1}\left[\text{exp}(X_{\left(t+h-k\right)}{}^{\prime }\beta )\times E(\text{exp}(\gamma ))+\frac{\nu -1}{2\nu }\right]\right)+\\ V(\text{exp}(\gamma ))\sum_{k=0}^{\infty }\rho^{2k}\text{exp}(X_{\left(t-k\right)}{}^{\prime }\beta )\text{exp}(X_{\left(t+h-k\right)}{}^{\prime }\beta ) \end{array}$

It is clear, from the above marginal moments that the ratio $\frac{V(Y_{t})}{E(Y_{t})}>1$ and hence the above model is suitable to model over-dispersion, in particular, severe over-dispersion.

We assume the following single-parameter models—Gamma and Normal distributions for the random effect *W* = exp(*γ*), given as:

1. $W∼Gamma(\frac{1}{c},c\text{exp}\left(\kappa^{2}/2\right))$, with *c* = exp(*κ*^2^) s.t
  $$\begin{array}{c} f_{W}\left(w\right)=\frac{\text{exp}{\left(-\{c\text{exp}\left(\kappa^{2}/2\right)\right\}}^{-1}w)w^{c^{-1}-1}}{{\left\{c\text{exp}\left(\kappa^{2}/2\right)\right\}}^{1/c}\Gamma \left(c^{-1}\right)}, \end{array}$$ (3)
  with *E*(*W*) = exp(*κ*^2^/2), *V*(*W*) = exp(2*κ*^2^)
2. *W* ∼ *Normal*(*κ*^2^)
  $$\begin{array}{c} f_{W}\left(w\right)=\frac{1}{\kappa \sqrt{(}2\pi )}\text{exp}\left(-\frac{{\left(w-\kappa^{2}\right)}^{2}}{2(\kappa^{2})}\right), \end{array}$$ (4)
  *E*(*W*) = *V*(*W*) = *κ*^2^,

where *κ*^2^ = *σ*^2^ + *ν*.

By assuming $\tilde{y_{t}}=\rho *\tilde{y_{t-1}}+\tilde{r_{t}}$, the conditional log-likelihood function for the set of observations (*y*_1_, *y*_2_, …, *y*_*t*_, …, *y*_*T*_) is written as:

$$\begin{array}{c} L=\int_{0}^{\infty }\prod_{t=2}^{T}f\left(\tilde{y_{t}}|\tilde{y_{\left(t-1\right)},\gamma }\right)g\left(\gamma \right)d\gamma \end{array}$$ (5)

where

f(yt˜|y(t-1),γ˜)=∑s=0min(yt˜,y(t-1)˜)(y(t-1)˜s)ρs(1-ρ)y(t-1)˜-s×frt˜(yt˜-s) (6)

From the above, the evaluation of the integrand in Eq (5) is performed using the numerical quadrature techniques using the *integral, fastGHQuad* packages in R and likelihood is thereon optimized using the *optim* function.

## Results

Further to the model construction and estimation procedures, in this section, some time-independent and time-variant covariates that could explain the fluctuations in the number of confirmed COVID-19 active cases in Mauritius have been considered. The following covariates are measured as from 18th March 2020 until 11th April 2020.

1. Time: is a time-dependent variable that indexes the *t*^*th*^ day.
2. Sanitary Curfew/Lockdown: is a binary variable with 1 or 0 where 0 indicates no sanitary curfew/lockdown and is considered as the reference variable (REF). In Mauritius, the sanitary curfew started from the 20th March 2020 and is prevalent until the 4th of May 2020. Several measures, like work access permits to allow normal functioning of essential service sectors, pre-defined visits to grocery stores for the purchase of essential commodities, mandatory COVID-19 screening of customers and strict adherence to social distancing in public places, have been put into place.
3. Num of Contravention Fines and Traffic Offences: The Mauritius Police Force (MPF) has instituted a number of barricades in restricted areas and road blockages, constantly and strictly being monitored. As of the end of April 2020, more than 8000 contravention fines, have been issued to lockdown rule breakers and based on the visual content circulating on the social media, several warnings have been given to people wandering without a particular purpose. This variable captures the number of contravention fines and traffic offences recorded since 20th March 2020.
4. Treatment: refers to adequate (1) or inadequate(0) (REF) availability of hydroxychloroquine tablets and personal protective equipment (PPE) which are extremely useful to curb the disease and treat COVID-19 patients on time.
5. Population Size: This is a time-dependent covariate that refers to the current number of people living in the country as from 18th March 2020. This figure varies with time as it is influenced by the number of deaths occurring from the mentioned date and the number of persons being repatriated.
6. Age Composition: This variable is a time-dependent binary indicator that demarcates whether the highest frequency of COVID-19 patients is above, equal (REF) or below 60 years.
7. Num of Quarantine Centers: This is a time-dependent variable that denotes the number of quarantine centers in Mauritius which kept on increasing as the situation of COVID-19 in Mauritius was worsening. As of 20th March 2020, 4 quarantine centres were instituted by the local health authorities but as of 11th April 2020, more than 12 quarantine centers, some of which were hotels cum self-isolation centers, have been put into place. As at date, the number of active cases is proportionate to the number of quarantine centres. With only 20 active cases and more than 200 health care workers under self-isolation, as of 27th April 2020, only 2 quarantine centres are under use.
8. Climatic Conditions: We propose to split this time-dependent variable as binary—fine (Sunny) and not fine (Rainy; Windy) (REF) weather. Some ongoing research shows that similar to seasonal outbreaks like influenza, measles, typhoid, the novel coronavirus is also seasonal as it originated in China, in December which is a cooler season. However, tropical countries have not been spared from this virus. Therefore, without real data over a number of seasons, it is challenging to conclude if the pandemic follows the same seasonal patterns as seen in more normal outbreaks [15, 16].
9. Safe Shopping Guidelines: Has there been any shopping guidelines imposed by the local authorities in Mauritius?—Yes:1; No:0 (REF). Indeed, as aforementioned, customers are authorised to go shopping in alphabetical order and a maximum of 30 mins per person has been allocated inside the supermarket. Individuals with normal body temperature are allowed inside the supermarket and social distancing should be respected. With the aim to prevent panic buying, a limited quantity of basic commodities are allowed to be purchased and to avoid consumer exploitation through unusual price rise, a list of controlled goods with regulated mark up has been implemented.
10. Sanitization Campaigns: is a binary variable that indicates whether there has been strong sanitization campaigns by the concerned health authorities—Yes:1; No:0 (REF). Indeed, screening exercises are being carried out in all public places. It is mandatory to wear protective masks and gloves during public outings. Areas where the highest number of COVID-19 cases were reported, have been disinfected. As per local authorities, COVID-19 screening on a larger scale is in the pipeline.
11. Sensitization campaigns: is a binary variable where ‘Yes’ indicates excessive marketing campaigns and ‘No’ (REF) means no campaigns have been done to sensitize the population—Yes:1; No:0 (REF). Stringent marketing campaigns are highly prevalent during this darkness of COVID-19. Many songs of hope in local and international languages, with the aim to communicate the seriousness of the situation and encourage the population to stay home, have been playing on social media. Slogans like “*stayhomestaysafe*” and “*BesafeMoris*” have been trending on social media to draw attention on the importance of confinement. In some strategic areas, the police force even addressed the public on important confinement-related matters using loudspeakers.

The results illustrate that the climatic conditions do not contribute quite significantly to the evolution of the COVID-19 in Mauritius, while the remaining variables do impact significantly on the number of COVID-19 cases. It can be noticed that the sanitary curfew/lockdown, sanitization and sensitization campaigns and safe shopping guidelines have helped to curb down, by a large extent, the number of COVID-19 cases in Mauritius. Hence, such preventive and pro-active measures, with the main focus on sanitization measures at grocery stores and in busy public places, should be maintained even after lifting lockdown orders. We can also note that the number of contravention fines and traffic offences tackled by the officers of the Mauritius Police Force have also helped in containing the virus as due to road blockages, the mobility of people are minimised leading to no public gatherings. The Treatment variable confirms availability of adequate hydroxychloroquine tablets to treat the number of COVID-19 cases in Mauritius. The number of quarantine centers have significantly helped in timely containment of the novel coronavirus, eliminating to a great extent the risk of spreading in the local community. As per the protocols set by the Ministry of Health and Wellness, as soon as people were repatriated to Mauritius, they were immediately self-isolated. Based on the numerical evidence, the age composition, in the local context, shows that people below 60 years of age has been more affected by COVID-19. The value of *σ*^2^ confirms the presence of some unobservable effects that are possibly contributing to the propagation of the virus. This could be linked to research findings in [17, 18] which showed that the SARS-CoV-2 is an aerosolized virus, particularly found in patients’ bathrooms, whilst others concluded that the small airborne particles could remain suspended in air and even flow up to six feet with the air current [19].

The results in Table 1 were obtained assuming the training dataset from 18th March 2020 to 11th April 2020. We use the above regression estimates or weights to forecast the number of infected COVID cases in Mauritius from 12th April 2020 to 23rd April 2020.

**Table 1:** Regression and variance estimates from COM-Poisson with Gamma and Normal distributed effects.

Factors	Estimates under	
	COM-Poisson Gamma	COM-Poisson Normal
Time	0.2314	0.2632
	(0.1151)	(0.1301)
Sanitary Curfew/Lockdown	-4.3512	-4.7092
	(0.2333)	(0.2556)
Num of Contravention/Traffic offence	-0.3616	-0.4202
	(0.1516)	(0.1662)
Treatment	-3.8912	-4.3310
	(0.2331)	(0.3010)
Population Size	1.3081	1.2811
	(0.1239)	(0.1287)
Age Composition (60)	1.2051	1.1975
	(0.0601)	(0.0598)
Num Quarantine Centres	-3.0881	-3.2112
	(0.1907)	(0.2010)
Climatic Conditions	0.0045	0.0010
	(0.1145)	(0.1335)
Safe Shopping Guidelines	-4.0551	-4.5610
	(0.2101)	(0.2236)
Sanitization Campaigns	-6.3214	-7.1211
	(0.1562)	(0.1572)
Sensitization Campaigns	-3.0234	-4.2014
	(0.1313)	(0.1334)
*ρ*	0.5609	0.5432
	(0.0601)	(0.0654)
*σ*^2^	0.8121	0.5408
	(0.0601)	(0.0654)
*ν*	0.2101	0.3091
	(0.0404)	(0.0434)

From Table 2, the Mean Squared Error (MSE) based on the proposed COM-Poisson Gamma mixture is computed as 3.8598 with relatively lower corresponding AIC, which can be viewed as satisfactorily adequate. In fact, the model slightly overstates the number of cases which is surely more beneficial and prudent to the policymakers. Local authorities, through provision of sufficient PPEs to “frontliners”, re-enforcement of the sanitary obligations and by upgrading the sanitization campaigns, can help to contain the virus more rapidly. Also, using these forecasts, the local health authorities can make reliable action plans, especially, regarding bed capacity in each COVID-19 hospitals in case of sudden increase in the number of COVID-19 cases in the future. Note that a similar prediction exercise was reported in Italy [20, 21] using an alternative forecasting method, but since not all its regions were under confinement and, most importantly, due to delay in policy making, the scenario went uncontrollable.

**Table 2:** AICs and MSEs based on Number of Infected Cases in Mauritius, from 12th April 2020 to 23rd April 2020.

Models	AIC	MSE
COM-Poisson Gamma	1232.1511	3.8598
COM-Poisson Normal	1456.0911	5.2101

## Discussion

In line with confinement measure, new feasible approaches including broadcasting of educational content on television for primary and secondary grade levels, work from home schemes, setting up of online purchasing platforms, provision of urgent court services via technological communication and a more safe and hygienic lifestyle have been adopted to limit socio-economical shocks. However, many individuals, especially working people and children, are exposed to this unprecedented stressful situation of unknown duration. It is proven that changes in work schedule and requirements and home schooling affect the psychological behaviours of most individuals [22, 23]. Here, right parenting skills and sustainable programmes by local stakeholders become crucial. For the tertiary sector, academics used intensively interactive online teaching platforms for virtual lecturing, as well as open source learning platforms for assignment practices.

For adequate food supply and a reasonable price mechanism on the local market, a controlled mark-up of 20 percent on some basic commodities and restrictions on quantity of basic groceries purchased, have been implemented. Local crop production is being encouraged through provision of diverse rebate schemes and reduction in the price of vegetable seeds.

Vulnerable families, where the head of the household are self-employed, or are informal sector workers or lead a Small and Medium Enterprise, are financially being assisted based on the new social protection schemes. The aforementioned economic activities have recently faced a drastic fall in performance due to the prolonged movement restrictions.

It is compulsory for any employee to be in possession of a Work Access Permit, when travelling to work. Misuse of the permit will lead to serious legal convictions.

Recently, the COVID-19 (Miscellaneous Provisions) Bill and the Quarantine Bill were implemented with foremost aim to drive the mauritian economy and to prevent any resurgence of the disease. Through setting up of a comprehensive set of strict sanitary measures, improvement in surveillance control and in the level of preparedness in the health care system, Mauritius remain on alert to stave off a second wave of SARS-CoV-2 in the country. The COVID-19 (Miscellaneous Provisions) bill brought in amendments to safeguard the fundamental rights of mauritians and most importantly the public health. In this context, it is believed that the findings from this study will act as a yardstick for policy makers when planning for preparedness measures and response strategies.

Every stakeholders, be it the individuals, the governmental or non-governmental organisations and the principal actors of the private sector, should bring in their collective capacities to work towards a “COVID-19-free” Mauritius. As of now, physical distancing is the new “norm” of the society.

## Conclusion

The results of this research revealed that several factors, the most significant being confinement measure, and least one being climatic conditions, affect the number of COVID-19 cases in Mauritius. It is worth mentioning that the proposed model adapts to the local data since the number of COVID-19 cases in Mauritius is mostly low integer-valued, hence, its usage is not generic as it depends on the nature and pattern of the COVID-19 data. Besides, some countries like, Seychelles, faces a zero-inflated pattern which implies the present model needs to be modified to accommodate for zero-inflated cases. However, as for Mauritius, this shall guide the policy makers to implement stricter preventive measures associated with legal obligations and economical tools to curb the pandemic on time and capitalise the Mauritian economy. The state of Mauritius should ensure that other jurisdictions like Rodrigues, Agalega and Chagos Archipelago, which lack high quality health care services are not affected by COVID-19. Research on COVID-19 should be ongoing due to pathological complexities of the novel coronavirus and unexplored area of research like seasonal effect on the spread of the virus [24].

## Supporting information

S1 Text (TXT)

S2 Text COVID-19 Data on Mauritius. (CSV)

S1 Appendix Proof. (PDF)
