# Supplementary material for: Studying the trend of the novel coronavirus series in Mauritius and its implications
Source: PLoS One. 2020 Jul 10;15(7):e0235730. doi: 10.1371/journal.pone.0235730 (PMC7351213; doi:10.1371/journal.pone.0235730)
Supplement: S1 Appendix — (PDF) [file pone.0235730.s003.pdf]

S1 Appendix. Proof

$$
\begin{aligned}
E(r_t) &= E\left[E(\widetilde{r_t})\right] \\
&= \exp(\boldsymbol{X_t}'\boldsymbol{\beta} + \gamma) \\
&= \exp(\boldsymbol{X_t}'\boldsymbol{\beta}) \times E(\exp(\gamma))
\end{aligned}
$$

$$
\begin{aligned}
V(r_t) &= E\left[V(\widetilde{r_t})\right] + V\left[E(\widetilde{r_t})\right] \\
&= E\left[\nu^{-1}(\widetilde{\mu_t} + \frac{\nu-1}{2\nu})\right] + V\left[\exp(\boldsymbol{X_t}'\boldsymbol{\beta} + \gamma)\right] \\
&= \nu^{-1}\left[\exp(\boldsymbol{X_t}'\boldsymbol{\beta}) \times E(\exp(\gamma)) + \frac{\nu-1}{2\nu}\right] + \exp(2(\boldsymbol{X_t}'\boldsymbol{\beta}) \times V(\exp(\gamma)))
\end{aligned}
$$

Using the relation

$$
\widetilde{y_t} = \sum_{k=0}^{\infty} \rho^k * \widetilde{r_{(t-k)}}
$$

We obtain

$$\begin{aligned} E(y_t) &= E\left[E(\widetilde{y_t})\right] \\ &= E\left[\sum_{k=0}^{\infty}\rho^k E(\widetilde{r_{(t-k)}})\right] \\ &= \sum_{k=0}^{\infty}\rho^k E(r_{(t-k)}) \\ &= E(\exp(\gamma))\sum_{k=0}^{\infty}\rho^k \exp(\boldsymbol{X_{(t-k)}}'\boldsymbol{\beta}) \end{aligned}$$

$$\begin{aligned} V(\widetilde{y_{ijt}}) &= E\left[V(\sum_{k=0}^{\infty}\rho^k * \widetilde{r_{(t-k)}}|\widetilde{r_{(t-k)}})\right] + V\left[E(\sum_{k=0}^{\infty}\rho^k * \widetilde{r_{(t-k)}}|\widetilde{r_{(t-k)}})\right] \\ &= E\left[\sum_{k=0}^{\infty}\rho^k(1-\rho^k)(\widetilde{r_{(t-k)}})\right] + V\left[\sum_{k=0}^{\infty}\rho^k(\widetilde{r_{(t-k)}})\right] \\ &= \sum_{k=0}^{\infty}\rho^k(1-\rho^k)E(\widetilde{r_{(t-k)}}) + \sum_{k=0}^{\infty}\rho^{2k}V(\widetilde{r_{(t-k)}}) \end{aligned}$$

assuming $Cov(\widetilde{r_{ij(t-k)}}, \widetilde{r_{ij(t-k')}}) = 0, k \neq k', k, k' \in Z^+$ Then,

$$\begin{aligned} V(y_t) &= E\left[V(\widetilde{y_t})\right] + V\left[E(\widetilde{y_t})\right] \\ &= \sum_{k=0}^{\infty}\rho^k(1-\rho^k)E(r_{(t-k)}) + \sum_{k=0}^{\infty}\rho^{2k}E(V(\widetilde{r_{(t-k)}})) + V(\sum_{k=0}^{\infty}\rho^k E(\widetilde{r_{(t-k)}}) \\ &= E(\exp(\gamma))\sum_{k=0}^{\infty}\rho^k(1-\rho^k)\exp(\boldsymbol{X_{(t-k)}}'\boldsymbol{\beta}) \\ &\quad + \sum_{k=0}^{\infty}\rho^{2k}\exp(-\phi)\left[\exp(\boldsymbol{X_{(t-k)}}'\boldsymbol{\beta}) \times E(\exp(\gamma_i)) + \frac{\nu-1}{2\nu}\right] \\ &\quad + \sum_{k=0}^{\infty}\rho^{2k}\exp(2\boldsymbol{X_{(t-k)}}'\boldsymbol{\beta}) \times V(\exp(\gamma)) \end{aligned}$$

assuming $Cov(\widetilde{r_{(t-k)}}, \widetilde{r_{(t-k')}}) = 0, k \neq k', k, k' \in Z^+$

$$\begin{aligned} Cov(y_t, y_{(t+h)}) &= E[Cov(\widetilde{y_t}, \widetilde{y_{(t+h)}})] + Cov[E(\widetilde{y_t}), E(\widetilde{y_{(t+h)}})] \\ &= \rho^h E[V(\widetilde{y_{(t+h)}})] + Cov[E(\widetilde{y_t}), E(\widetilde{y_{(t+h)}})] \\ &= \rho^h\left(\sum_{k=0}^{\infty}\rho^k(1-\rho^k)E(\exp(\gamma))\exp(\boldsymbol{X_{(t+h-k)}}'\boldsymbol{\beta})\right) \\ &\quad + \rho^h\left(\sum_{k=0}^{\infty}\rho^{2k}\nu^{-1}\left[\exp(\boldsymbol{X_{(t+h-k)}}'\boldsymbol{\beta}) \times E(\exp(\gamma)) + \frac{\nu-1}{2\nu}\right]\right) \\ &\quad + V(\exp(\gamma))\sum_{k=0}^{\infty}\rho^{2k}\exp(\boldsymbol{X_{(t-k)}}'\boldsymbol{\beta})\exp(\boldsymbol{X_{(t+h-k)}}'\boldsymbol{\beta}) \end{aligned}$$
